# Characterization of Phthalate Exposure among Pregnant Women Assessed by Repeat Air and Urine Samples

**DOI:** 10.1289/ehp.10749

**Published:** 2008-01-15

**Authors:** Jennifer J. Adibi, Robin M. Whyatt, Paige L. Williams, Antonia M. Calafat, David Camann, Robert Herrick, Heather Nelson, Hari K. Bhat, Frederica P. Perera, Manori J. Silva, Russ Hauser

**Affiliations:** 1 Department of Environmental Health, Harvard School of Public Health, Boston, Massachusetts, USA; 2 Columbia Center for Children’s Environmental Health, Mailman School of Public Health, Columbia University, New York, New York, USA; 3 Department of Biostatistics, Harvard School of Public Health, Boston, Massachusetts, USA; 4 National Center for Environmental Health, Centers for Disease Control and Prevention, Atlanta, Georgia, USA; 5 Southwest Research Institute, San Antonio, Texas, USA

**Keywords:** creatinine, indoor air, personal air, phthalates, pregnancy, specific gravity, urinary metabolites, variability

## Abstract

**Background:**

Although urinary concentrations of phthalate metabolites are frequently used as biomarkers in epidemiologic studies, variability during pregnancy has not been characterized.

**Methods:**

We measured phthalate metabolite concentrations in spot urine samples collected from 246 pregnant Dominican and African-American women. Twenty-eight women had repeat urine samples collected over a 6-week period. We also analyzed 48-hr personal air samples (*n* = 96 women) and repeated indoor air samples (*n* = 32 homes) for five phthalate diesters. Mixed-effects models were fit to evaluate reproducibility via intraclass correlation coefficients (ICC). We evaluated the sensitivity and specificity of using a single specimen versus repeat samples to classify a woman’s exposure in the low or high category.

**Results:**

Phthalates were detected in 85–100% of air and urine samples. ICCs for the unadjusted urinary metabolite concentrations ranged from 0.30 for mono-ethyl phthalate to 0.66 for monobenzyl phthalate. For indoor air, ICCs ranged from 0.48 [di-2-ethylhexyl phthalate (DEHP)] to 0.83 [butylbenzyl phthalate (BBzP)]. Air levels of phthalate diesters correlated with their respective urinary metabolite concentrations for BBzP (*r* = 0.71), di-isobutyl phthalate (*r* = 0.44), and diethyl phthalate (DEP; *r* = 0.39). In women sampled late in pregnancy, specific gravity appeared to be more effective than creatinine in adjusting for urine dilution.

**Conclusions:**

Urinary concentrations of DEP and DEHP metabolites in pregnant women showed lower reproducibility than metabolites for di-*n*-butyl phthalate and BBzP. A single indoor air sample may be sufficient to characterize phthalate exposure in the home, whereas urinary phthalate biomarkers should be sampled longitudinally during pregnancy to minimize exposure misclassification.

Phthalates are a class of synthetic compounds used widely in polyvinyl chloride plastics, in cosmetics, and in building materials. Biomonitoring data suggest that > 75% of the U.S. population is exposed to phthalates, including di-(2-ethylhexyl) phthalate (DEHP), di-*n*-butyl phthalate (DnBP), di-isobutyl phthalate (DiBP), butylbenzyl phthalate (BBzP), and diethyl phthalate (DEP) (Silva et al. 2004a). When urinary concentrations of secondary metabolites of DEHP [e.g., mono-(2-ethyl-5-oxohexyl) phthalate (MEOHP), mono-(2-ethyl-5-hydroxyhexyl) phthalate (MEHHP)] are measured, this estimate increases to 95% (Kato et al. 2004). It is assumed that the general population is exposed to some phthalates mainly through ingestion of foods contaminated during processing and packaging (Schettler 2006; Wormuth et al. 2006). Dermal contact with products containing phthalates, inhalation of indoor air, and inhalation or ingestion of household dust also contribute to widespread exposures.

Once a pregnant woman is exposed, phthalates can cross the placenta and enter fetal circulation (Mose et al. 2007). Phthalates have been detected in physiologically relevant compartments within pregnant women and the developing fetus, such as maternal urine (Adibi et al. 2003; Swan et al. 2005), cord blood (Latini et al. 2003), meconium (Kato et al. 2006), placenta (Mose et al. 2007), and amniotic fluid (Silva et al. 2004b).

Previous studies on the reproducibility of phthalate metabolite concentrations in urine samples among nonpregnant individuals have shown a wide range of estimates of within-person variability (Fromme et al. 2007; Hauser et al. 2004; Hoppin et al. 2002; Teitelbaum et al. 2007), leading to concerns regarding the approach of relying on a single sample to characterize exposure. In addition, these studies may not accurately represent what happens during pregnancy when a woman’s physiology is dramatically altered.

The primary aim of the current study was *a*) to use biomarkers of exposure (i.e., metabolites measured in urine) to evaluate variability in phthalate concentrations in pregnant women; and *b*) to evaluate variability in measures of phthalates in their external environment (i.e., indoor air). As a secondary aim, we evaluated correlations between phthalate metabolite concentrations measured in maternal and newborn urine. Finally, we evaluated the correlation between phthalate levels in personal/indoor air and urinary metabolite concentrations.

## Methods

### Study population

Subjects were recruited into the Columbia Center for Children’s Environmental Health (CCCEH) cohort at prenatal clinics located in three New York City neighborhoods in Northern Manhattan and the South Bronx. To be eligible for enrollment, subjects had to reside in the study area for at least 1 year, receive their first prenatal visit by the 20th week of pregnancy, and be free of diabetes, hypertension, and known HIV and drug or alcohol abuse (Perera et al. 2006; Whyatt et al. 2003). A variability study using a repeated-measures design was carried out in a subset of 102 women from the full CCCEH cohort (Whyatt et al. 2007). Variability study subjects were not employed outside the home. At the time of enrollment, all subjects completed a prenatal questionnaire. Information was also abstracted from prenatal and delivery medical records. The CCCEH study and the substudies were approved by the institutional review boards of Columbia University and the Centers for Disease Control and Prevention (CDC) and the Harvard School of Public Health Human Subjects Committee. Written informed consent was obtained from all study subjects.

### Phthalate measures

#### Sampling design

During the third trimester of pregnancy, women (*n* = 96) were asked to wear a small backpack holding a personal ambient air monitor during the daytime hours for 2 consecutive days and to place the monitor near their beds at night. Over this period, the personal air sampling pumps operated continuously at 4 L/min, collecting particles ≤ 2.5 μm in diameter on a precleaned quartz microfiber filter and collecting semivolatile vapors and aerosols on a polyurethane foam (PUF) cartridge backup. At the end of the 48-hr monitoring period, all women gave a spot urine sample, which we refer to as 48-hr monitoring. Urine samples were also collected in the hospital from a subset of mothers (*n* = 16) and from their newborns (*n* = 19) 1 day after delivery. The newborn urine samples were collected by attaching urine-collection bags to the babies.

The variability study was carried out on a subset of subjects (*n* = 32) who completed the 48-hr personal monitoring. Indoor air monitors placed in the women’s apartments ran continuously for 2 weeks. As previously described by Whyatt et al. (2007), monitoring was conducted using a pump with a 0.5 L/min flow-rate attached to a similar PUF sampler. At the end of each 2-week period, the air sampler and battery were replaced and the subject gave a spot urine sample. The indoor air sampling began at 31.0 ± 1.7 (mean ± SD) weeks of gestation, and the urine collection began at 33.0 ± 1.7 weeks of gestation. The indoor air monitoring and urine collection continued until the women went into labor and will be referred to as the 6-week monitoring. When possible, we combined the 48-hr and 6-week monitoring periods to give a total of 8 weeks of observation.

#### Urinary metabolite measures

All urine samples were analyzed at the National Center for Environmental Health of the CDC for four phthalate metabolites: mono-ethyl phthalate (MEP), mono-*n*-butyl phthalate (MnBP), mono-benzyl phthalate (MBzP), and mono-2-ethylhexyl phthalate (MEHP). As analytical methods and knowledge of phthalate metabolism improved, the panel of urinary metabolites increased, and most participants also had measures for five additional metabolites: MEOHP, MEHHP, mono-2-ethyl-5-carboxypentyl phthalate (MECPP), mono-isobutyl phthalate (MiBP), and mono-3-carboxypropyl phthalate (MCPP). The 48-hr study urine samples were analyzed in four separate batches during 2001–2006. The variability study urine samples were all analyzed in the 2006 batch.

The analytical approach for measuring urinary phthalate metabolites involved enzymatic deconjugation of the metabolites from their glucuronidated form, solid-phase extraction, separation with high-performance liquid chromatography, and detection by isotope-dilution tandem mass spectrometry (Blount et al. 2000; Kato et al. 2005; Silva et al. 2003; Silva et al. 2004c). To monitor for accuracy and precision, each analytical run included—together with unknown samples—calibration standards, reagent blanks, and quality control materials of high and low concentrations. The limits of detection (LODs), which varied slightly depending on the method used, were in the low nanogram-per-milliliter range. Concentrations < LOD were set to one-half the LOD for calculations. Creatinine concentration was measured using an enzymatic reaction on a Roche Hitachi 912 chemistry analyzer (Roche Hitachi, Basel, Switzerland). Metabolite concentrations were creatinine-adjusted to give micrograms per gram creatinine. As an alternative to creatinine, specific gravity was measured in the variability study samples. Methods for specific gravity measurement are described elsewhere (Hauser et al. 2004).

#### Personal and indoor air analysis

The personal and indoor air samples were analyzed at the Southwest Research Institute for five phthalates: DEHP, DnBP, BBzP, DiBP, and DEP. Methods are described elsewhere (Rudel et al. 2003). After being stored and shipped at −4°C, the PUF and filter were soxhlet-extracted with 6% diethyl ether in hexane and concentrated to 10 mL, of which an aliquot was used for phthalate analysis. Gas chromatography (GC)/mass spectrometry was performed using an Agilent 6890 GC equipped with an Agilent 5973 Mass Selective Detector (Agilent Technologies Inc., Santa Clara CA) in selected ion monitoring mode. Two deuterated phthalates were used as internal standards for quantitation.

Phthalates were measured in blank PUF cartridges to estimate levels of potential phthalate contamination in the sampling and analysis process. If phthalate amounts in the blanks were > LOD, the samples were flagged. To obtain the concentration of phthalate per cubic meter of air, we divided the extract value by the total volume (in cubic meters) of air pulled through the pump during the sample period. Although mean phthalate levels in personal air samples were at least an order of magnitude higher than in air matrix blanks, a few air sample amounts of BBzP and DEHP were lower than the maximum air matrix blank amount for that phthalate.

### Statistical analysis

Phthalate diester levels in air (micrograms per cubic meter) and phthalate metabolites in urine (nanograms per milliliter) are reported as percentiles, and as geometric means with corresponding 95% confidence intervals (CIs). %MEHP3, the ratio of MEHP to three DEHP metabolites (MEHP, MEOHP, and MEHHP), was used as a phenotypic marker of DEHP metabolism (Hauser et al. 2007). %MEHP4 is the ratio of MEHP to four DEHP metabolites (MEHP, MEOHP, MEHHP, and MECPP). Using geometric means and 95% CIs, we compared concentrations measured in the CCCEH study population with those measured in the National Health and Nutrition Evaluation Survey (NHANES) [CDC/National Center for Health Statistics (NCHS) 1999–2000, 2001–2002]. We used publicly accessible urinary concentration data from NHANES 1999–2000 and 2001–2002 for eight metabolites to estimate the mean concentrations in U.S. females between the ages of 18 and 40 years. Additionally, we calculated the geometric mean concentration and 95% CI for U.S. pregnant females (*n* = 209 for MEHP, MEP, and MBzP; *n* = 104 for MEOHP, MEHHP, MnBP, MiBP, and MCPP). Given that the NHANES sample was nonrandom, we used the recommended methods to correctly estimate variances (NCHS 2007).

To evaluate correlations between untransformed concentrations of phthalates in urine (metabolites), indoor air, and personal air, we used Spearman correlations with a Fisher *Z* transformation to estimate 95% CIs. In cases of multiple samples per subject, we used a geometric mean to summarize all values for that subject. When modeling variability, we applied a logarithmic transformation to the air and urine phthalate measurements to better approximate a normal distribution. Mixed-effects models were fit to estimate the temporal variability in phthalate concentrations and to estimate the intraclass correlation coefficients (ICCs). We chose the most appropriate covariance structure by comparing Akaike information criterion (AIC) values between a covariance that assumes a constant correlation between any pair of measurements made on the same subject versus a first-order autoregressive structure, which assumes that measurements on the same subject taken closer in time are more highly correlated than those taken further apart. An ICC, the ratio of between-subject variance to total variance, is a measure of reproducibility of a biomarker sampled from the same group of individuals over time, and ranges from 0 (no reproducibility) to 1 (perfect reproducibility; i.e., 100% of the variance is due to between-subject differences) (Rosner 2000).

The sensitivity and specificity of using a single urine sample per woman to classify her exposure to phthalates as low or high, compared with using three to five samples per subject, was estimated by randomly selecting a single sample from among each woman’s repeated samples collected over all 8 weeks. The metabolite concentrations (nanograms per milliliter) for the single sample were compared to the NHANES geometric mean (nanograms per milliliter) for that metabolite calculated for U.S. women of reproductive age and classified as below (low) or above the geometric mean (high). The geometric mean of a woman’s repeat samples was considered to reflect her “true” exposure and was similarly classified as low or high relative to the NHANES geometric mean for that metabolite. For each woman, the single selected sample was compared to the geometric mean of all her remaining samples (excluding the selected sample) in terms of low versus high exposure, and the sensitivity and specificity were calculated. This process was repeated 1,000 times, generating 1,000 estimates of sensitivity and specificity. We report the empirical estimates of the median sensitivity and specificity and empirical CIs for each metabolite. We used SAS, version 9.1 (SAS Institute Inc., Cary, NC) for all statistical analyses. We used Microsoft Excel 2003 SP2 (Microsoft Corporation, Redmond, WA) to generate graphics.

## Results

### Background characteristics of the study sample

The demographics of our study sample, shown in Table 1, reflect the demographics of the overall CCCEH cohort. The mean age was 25.6 years; 74% of subjects were Dominican; 74% had an education at or below high school level; and 62% were never married. The subjects in the variability study were similar in age and marital status and were more likely to have a lower educational level (81% had a high school education or less).

### Urinary phthalate metabolites in pregnant women and newborns

The distributions of the nine urinary phthalate metabolite concentrations among pregnant women are summarized in Table 2. All urinary metabolites were detected in the pregnant women at 100% frequency, except for MEHP (85%) and MCPP (89%). Geometric mean concentrations of two urinary metabolites (MnBP, MiBP) were higher in the CCCEH participants than in the NHANES females of reproductive age (18–40 years) (Table 2). In pregnant women, the geometric means were significantly higher in the CCCEH subjects than in the NHANES participants for MnBP (37.5 vs. 19.6 ng/mL) and for MiBP (9.5 vs. 2.5 ng/mL). On average, the CCCEH subjects had a significantly lower %MEHP than the NHANES pregnant females (11% vs. 17%).

**Figure 1:**
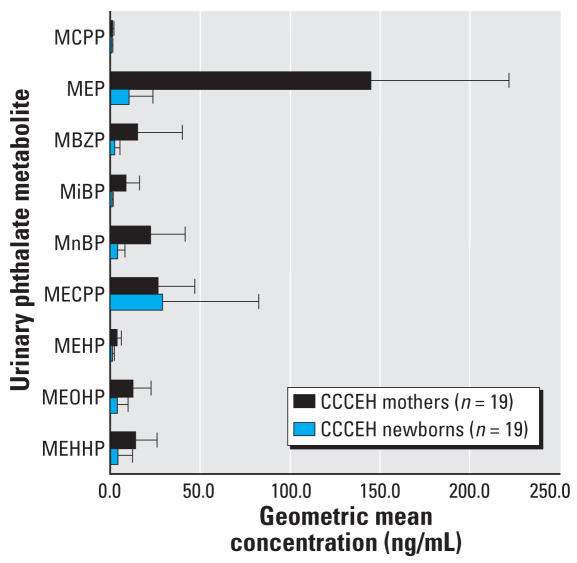
Urinary profile of phthalate metabolite concentrations in pregnant women and their newborns. Error bars represent upper bounds of 95% CIs.

Comparisons between concentrations in mothers and their newborns are illustrated in Figure 1. In the newborns, the detection frequencies were 42% (MEHP), 68% (MCPP, MEOHP, and MEHHP), 89% (MBzP and MnBP), 99% (MEP), and 100% (MECPP). Most metabolite concentrations in the newborns were consistently lower than maternal concentrations based on the geometric mean. However, the median concentration of MECPP was higher in the newborns than in the mothers (56.9 vs. 36.1 ng/mL).

**Figure 2:**
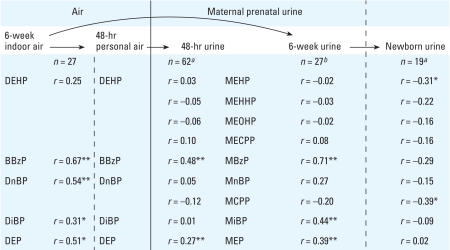
Summary of Spearman coefficients of correlation between phthalate concentrations measured in environmental and biologic matrices. ^***a***^Unadjusted for urinary dilution (data not available). ^***b***^Adjusted for specific gravity. *p < 0.20. **p < 0.05.

We found no correlation between phthalate metabolite concentrations measured in urine samples collected from mothers and their newborns approximately 1 day after delivery. For three metabolites, there was a suggestive inverse correlation between the geometric mean of 19 mothers’ samples (two to five urine samples collected over 8 weeks before delivery) with their newborns’ urinary metabolites measured 1 day after delivery (MEHP, *r* = −0.31, *p* = 0.19; MCPP, *r* = −0.39, *p* = 0.09; MBzP, *r* = −0.29, *p* = 0.22) (Figure 2).

### Phthalate measurements in personal and indoor air

Concentrations of five phthalate diesters measured in personal air are summarized in Table 3. All five phthalates were detected at 100% frequency in the air samples. The geometric mean values were higher for personal air compared with indoor air. There was overlap in the CIs for all phthalates except DEHP, which was significantly higher by 2-fold in the personal air samples. When we limited the analysis to subjects who had both a personal air and indoor air sample (*n* = 27), there was a positive correlation between 48-hr personal air and the average indoor air levels over the 8 weeks of sampling for all five phthalate diesters, estimated as 0.54 for DnBP (*p* = 0.002), 0.67 for BBzP (*p* < 0.0001), 0.51 for DEP (*p* = 0.005), 0.31 for DiBP (*p* = 0.11), and 0.25 for DEHP (*p* = 0.21) (Figure 2).

### Variability study: urinary phthalate metabolites

For the urine variability analysis, we excluded 3 women with only one urine sample and 1 woman with missing urinary creatinine values. Of the remaining 28 subjects, 12 women had two samples and 16 women had three or four samples. We were limited to samples collected over the 6-week monitoring period because of missing data on urinary dilution for the 48-hr monitoring sample. Onset of labor was the primary reason that urine samples were unavailable for some subjects at weeks 4 and 6, which correspond approximately to weeks 37 and 39 of gestation.

The ICCs for the nine metabolites in urine ranged from 0.30 to 0.66 without adjustment for creatinine, and decreased to 0.21–0.65 with creatinine adjustment (Table 4). MEP and the metabolites of DEHP had the lowest reproducibility, with ICCs ranging from 0.30 to 0.36. %MEHP was a stable measure within a woman during 6 weeks in late pregnancy, with an ICC of 0.64 for %MEHP3 and 0.60 for %MEHP4. We also compared ICCs calculated with adjustment for specific gravity in a subset of 22 subjects sampled over the same time period. For the DEHP and DEP metabolites, the specific gravity ICC estimates were higher than those for creatinine-adjusted metabolites but lower than the unadjusted estimates. For the DnBP and BBzP metabolites, the specific gravity estimates were higher than both the unadjusted and the creatinine-adjusted estimates (data not shown). The covariance structure that assumes a constant correlation between any two measurements on a single subject yielded a better model fit (lower AIC value) and was applied in all mixed-effects models. We did not detect significant temporal trends in metabolite levels over the 6-week period.

We evaluated the sensitivity and specificity of characterizing exposure based on a single sample compared with all available samples using women who had three to five repeated urine samples over 8 weeks (*n* = 26) (Table 5). The probability of correctly classifying a woman as having high exposure based on a single randomly selected urine sample, if she truly was in a high exposure category based on all her urine samples (i.e., sensitivity), ranged from 0.50 (MiBP) to 0.74 (MCPP). The probability ranged from 0.43 (MEP) to 0.95 (MiBP) of correctly classifying a woman as having low exposure if she truly was in a low-exposure category (i.e., specificity).

### Variability study: phthalate measures in air

For the 32 women in the 6-week monitoring study, 6 provided two indoor air samples each, 11 women provided three samples each, and 15 women provided four samples each. Within a woman’s home, the indoor air phthalate levels were more stable over time than were her urinary phthalates. The ICCs were 0.61 for DEP, 0.54 for DiBP, 0.59 for DnBP, 0.48 for DEHP, and 0.83 for BBzP.

### Association between phthalate levels measured in air and urine

We calculated estimated Spearman correlation coefficients between phthalate levels in paired indoor air and urine samples collected over 6 weeks in late pregnancy (*n* = 27). These correlations were compared to paired 48-hr personal air and urine samples (*n* = 62) (Figure 2). After adjustment for specific gravity, which tended to yield stronger correlations than after adjustment for creatinine, we saw a significant association between BBzP in indoor air and MBzP in urine (*r* = 0.71, *p* < 0.0001) and between BBzP in personal air and MBzP in urine (*r* = 0.48, *p* < 0.0001). The correlation between DiBP in indoor air and MiBP in urine was weaker (*r* = 0.44, *p* = 0.02). There were significant associations between DEP in indoor air and MEP in urine (*r* = 0.39, *p* = 0.04) and DEP in personal air and MEP in urine (*r* = 0.27, *p* = 0.04). No associations were detected between DEHP or DnBP and their respective metabolites.

## Discussion

In a small sample of pregnant women, the reproducibility of urinary phthalate metabolite concentrations measured over a period of 6–8 weeks late in pregnancy was low to moderate. Reproducibility can be ranked in the following order: MEP (0.30), DEHP metabolites (mean ICC = 0.35), DnBP metabolites (mean ICC = 0.58), %MEHP3 and %MEHP4 (mean ICC = 0.62), and MBzP (0.64). The proposed measure of interindividual differences in metabolism and excretion (%MEHP) was more stable over time within a pregnant woman than the corresponding DEHP metabolites by approximately 2-fold. The CCCEH subjects had significantly higher mean urinary concentrations of MiBP (3-fold) and MnBP (43%) than U.S. females of reproductive age.

An ICC of 0.40 has been proposed as a cutoff for sufficient reproducibility in a biomarker to justify its use in an epidemiologic analysis (Rosner 2000) and has been cited in previous reports on the reproducibility of phthalate urinary metabolites (Hauser et al. 2006; Rosner 2000; Teitelbaum et al. 2007). However, this cutoff may be arbitrary and could still allow substantial misclassification that would bias an effect estimate toward the null. According to a simulation study conducted by de Klerk (1989), an exposure variable with an ICC of 0.42 would be associated with 32% attenuation in the estimated relative risk due to exposure misclassification, which is clearly undesirable. We observed ICCs < 0.50 for MEP and DEHP metabolites, suggesting that within-subject variability may be of greater magnitude than between-subject variability. Thus, studies relying on a single sample per subject may have unreliable effect estimates, and ideally we would recommend using repeated measures taken over the entire course of the pregnancy.

Within-subject variability in phthalate concentrations measured in indoor air during the same 6-week period was lower than that in urine, suggesting that exposures to phthalates are relatively constant within the home. Of the phthalates measured, DEHP has the lowest volatility (Wormuth et al. 2006), which might explain its lower concentrations in indoor air. Other studies have shown air concentrations of DEHP to be low, whereas household dust concentrations are consistently high (Bornehag et al. 2004; Rudel et al. 2003). Poor reproducibility for DEHP might mean the air concentrations are dependent on dust concentrations at the time of sampling, which could be associated with intermittent activities such as cleaning and moving furniture. BBzP was the most stable phthalate measured in indoor air.

To date, four separate studies have reported estimates of reproducibility of urinary phthalate metabolites (Fromme et al. 2007; Hauser et al. 2004; Hoppin et al. 2002; Teitelbaum et al. 2007). ICC estimates vary considerably, given the differences in study design, exposure patterns, age, and underlying physiology of subjects. We compared the rank orders of the estimates between studies. Hoppin et al. (2002), measured variability over a short period of 2 days and found all metabolites to be highly reproducible, and Hauser et al. (2004) found MEP to be the most reproducible, thus suggesting a different exposure pattern. In contrast, in the present study, we saw a trend by which the DnBP metabolites and the BBzP metabolites were consistently the most reproducible and the DEHP and DEP metabolites were the least reproducible within a person over time. This comparison reinforces the importance of characterizing inter- and intrasubject variability for different populations.

Depending on the research question, investigators may choose to group subjects by tertiles, quintiles, or even into low- and high-exposure categories. When relying on a single urine sample, this may reduce exposure misclassification because of within-subject variability. In our analysis of sensitivity and specificity, we found a similar trend as with the ICCs. DEP (0.43) and DEHP metabolites averaged (0.64) had the lowest specificity, whereas BBzP (0.73) and DnBP metabolites averaged (0.80) had the highest.

The observation that creatinine adjustment actually increased within-person variability and reduced reproducibility in urinary concentrations of phthalate metabolites was unexpected and inconsistent with other studies (Fromme et al. 2007; Hoppin et al. 2002). This difference may be explained by physiologic changes in late pregnancy that could alter creatinine production and/or excretion. Creatinine excretion on average increases by 30% in a pregnant woman, as does kidney size (Williams 2005). During the third trimester, however, there is a precipitous drop in the renal blood flow rate that could alter creatinine excretion (Williams 2005). For these reasons, creatinine may vary independently of phthalate excretion late in pregnancy. We compared variability in creatinine and specific gravity for 24 women who provided two to four repeat urine samples over 4–6 weeks. We found that creatinine had a lower ICC of 0.36 compared to specific gravity, which had an ICC of 0.58. Specific gravity, which is a measure of urine turbidity, has been proposed as a more appropriate method for adjusting phthalate concentrations (Hauser et al. 2004). In the case of pregnant women, we also propose that alternative methods be explored.

The presence of MECPP in 100% of the newborn samples and the fact that it was at the highest concentration suggests that the newborns were exposed to DEHP *in utero*, during the labor and delivery process, and/or within the first 24 hr after delivery. MECPP, which is largely in its free, unglucuronidated form in urine and has the longest elimination half-life of the DEHP metabolites examined, may be an appropriate biomarker of cumulative DEHP dose (Koch et al. 2006; Silva et al. 2006). The inverse correlations and higher maternal versus newborn concentrations that we observed for some of the metabolites may indicate that placental transporters are involved in actively shuttling phthalate metabolites out of fetal circulation, as it has already been established that they diffuse passively into fetal circulation in rodents (Saillenfait et al. 1998; Singh et al. 1975). However, we are not aware of data to support this hypothesis.

The correlations between phthalates in indoor air and urine partly confirmed our previous report (Adibi et al. 2003). Differences between that study and the present one may be due to differences in statistical power, sampling variability, confounding, temporal trends in exposure, and possibly batch effects in the laboratory analyses. Air concentrations of BBzP, which is commonly used in artificial rubbers, spray paints, and furniture coverings, were correlated significantly with urinary concentrations of MBzP and at the highest magnitude of those measured. Even though we had repeat measures of both indoor air and urine increasing our power to detect an association, we were still limited by a small sample size of 27.

## Conclusion

In the present study, we found urinary phthalate metabolite concentrations to be moderately to highly variable in a small sample of pregnant women sampled over 6 weeks late in pregnancy, whereas indoor air concentrations were more stable during the same period. The variability that we observed could be in part due to changes in exposure and/or physiologic changes in pregnancy, which alter metabolism and excretion of phthalates. As proposed in previous studies (Hauser et al. 2007), %MEHP proved to be a stable measure within a person over time and should be explored as a measure of phenotypic differences in metabolism and excretion. Our findings also suggest that creatinine adjustment might not be the optimal method of urinary dilution adjustment for subjects sampled late in pregnancy. Future research should be directed at increasing the number of urine samples collected and the number of intervals between samples over the duration of the pregnancy to reduce misclassification in measures of phthalate exposure. This will strengthen our ability to evaluate risks to the mother and the fetus associated with prenatal phthalate exposures.

## Figures and Tables

**Table 1:** Demographic characteristics of CCCEH subjects with phthalate measurements.

Characteristic	Full study (*n* = 246)	48-hr monitoring (*n* = 96)	6-week monitoring (*n* = 32)
Age [years (mean ± SD)]	25.6 ± 4.6^a^	25.1 ± 4.3^b^	26.0 ± 5.0
Gestational age [weeks (mean ± SD)]	39.2 ± 1.9^c^	39.3 ± 1.6^d^	39.2 ± 3.1^e^
Dates of enrollment [years (range)]	1999–2005	2000–2005	2002–2004
Years of education			
Missing	3 (1)	3 (3)	0
< High school	91 (37)	38 (41)	17 (53)
High school or GED	89 (37)	33 (36)	9 (28)
> High school	63 (26)	22 (23)	6 (19)
Ethnicity			
Missing	4 (2)	3 (3)	0
Dominican/Dominican American	179 (74)	75 (81)	25 (78)
African American	63 (26)	18 (19)	7 (22)
Marital status			
Missing	4 (2)	3 (3)	0
Never married	152 (62)	61 (66)	21 (66)
Married	76 (31)	30 (32)	8 (25)
Separated, widowed, or divorced	14 (6)	2 (2)	3 (9)
Household income (US$)			
Missing	31 (13)	14 (15)	6 (19)
< 10,000	94 (44)	37 (45)	11 (42)
10,000–30,000	96 (47)	35 (43)	14 (54)
> 30,000	25 (9)	10 (12)	1 (4)

GED, General Educational Development. Values are no. (%) except where indicated.

a Four missing.

b Three missing.

c Thirty-three missing.

d Five missing.

e Two missing.

**Table 2:** Urinary phthalate metabolite concentrations (ng/mL) in Dominican-American and African-American pregnant women living in New York City, and comparison with a U.S. population–based sample (NHANES).

				Percentile						
Phthalate diester	Phthalate metabolite	No.	Percent > LOD	5th	25th	50th	75th	95th	CCCEH (*n* = 246) [GM (95% CI)]	NHANES^a^ [GM (95% CI)]
DEP	MEP	246	100	36.8	103	202	481	2,753	232 (199–272)	235 (200–277)
DEHP	MEHP	246	85	0.5	2.2	4.8	13.6	46.8	4.8 (4.0–5.8)	4.5 (4.0–5.0)
	MEOHP	221	100	2.8	9.4	17.5	33.6	107.6	18.2 (15.6–21.3)	13.8 (10.4–18.3)
	MEHHP	221	100	2.5	10.4	19.9	43.4	149.6	20.2 (17.2–23.6)	19.8 (14.9–26.2)
	MECPP	220	100	6.2	20.5	37.1	81.2	232.2	38.3 (33.3–44.0)	NA
	%MEHP3	221	NA^b^	3%	7%	12%	18%	28%	11% (10–12)	13% (11–14)
	%MEHP4	220	NA^b^	1%	4%	7%	10%	17%	6% (5–6)	NA
DnBP/DiBP	MnBP	246	100	7.3	23.0	35.5	70.1	174.9	37.5 (33.3–42.2)	21.5 (17.1–27.2)
	MiBP	221	100	1.9	5.4	10.2	17.1	36.1	9.5 (8.4–10.8)	3.0 (2.5–3.5)
DnOP/DBP	MCPP	220	89	0.3	1.1	2.0	3.8	8.0	1.9 (1.6–2.2)	2.6 (2.1–3.3)
BBzP	MBzP	246	100	1.7	8.0	17.2	43.6	146.8	17.5 (14.8–20.7)	16.5 (14.2–19.2)

DnOP, di-*n*-octyl phthalate; GM, geometric mean; NA, not applicable.

a Females 18–40 years of age; *n* = 853 for metabolites measured in 1999–2002 (MEP, MEHP, MBzP); *n* = 437 for metabolites measured in 2001–2002 only (MEOHP, MEHHP, MnBP, MiBP, MCPP).

b Values < LOD were set to 0.5 × LOD and used in %MEHP calculation.

**Table 3:** Phthalate concentrations (μg/m^3^) in 48-hr personal air samples of pregnant Dominican-American and African-American women living in New York City compared with geometric means of indoor air.

			Percentile					Geometric mean (95% CI)	
Phthalate diester	No.	Percent > LOD	5th	25th	50th	75th	95th	Personal air	Indoor air (*n* = 32)
DEHP	96	100	0.07	0.11	0.19	0.29	0.49	0.18 (0.16–0.21)	0.09 (0.08–0.10)
DnBP	96	100	0.19	0.32	0.48	0.63	1.04	0.45 (0.41–0.51)	0.38 (0.33–0.45)
BBzP	96	100	0.01	0.02	0.04	0.13	0.27	0.05 (0.04–0.06)	0.03 (0.02–0.04)
DiBP	96	100	0.17	0.33	0.50	0.77	1.43	0.50 (0.44–0.57)	0.45 (0.38–0.53)
DEP	96	100	0.84	1.47	2.33	3.36	5.06	2.15 (1.92–2.41)	1.66 (1.38–2.00)

**Table 4:** Variance in log-transformed urinary phthalate metabolite concentrations in pregnant women sampled over 6 weeks in late pregnancy (*n* = 28; 2–4 repeats).

		Log-transformed concentration	
Phthalate diester	Phthalate metabolite	Unadjusted	Creatinine adjusted
DEHP	MEHP		
	ICC	0.35	0.25
	Subject (SE)	0.79 (0.39)	0.53 (0.34)
	Residual (SE)	1.46 (0.31)	1.56 (0.34)
	MEOHP
	ICC	0.34	0.22
	Subject (SE)	0.58 (0.28)	0.36 (0.25)
	Residual (SE)	1.14 (0.24)	1.26 (0.27)
	MEHHP
	ICC	0.36	0.23
	Subject (SE)	0.69 (0.33)	0.41 (0.28)
	Residual (SE)	1.23 (0.26)	1.41 (0.30)
	MECPP
	ICC	0.33	0.21
	Subject (SE)	0.53 (0.27)	0.31 (0.23)
	Residual (SE)	1.08 (0.23)	1.16 (0.25)
	%MEHP3
	ICC	0.64	NA^a^
	Subject (SE)	0.39 (0.13)	
	Residual (SE)	0.22 (0.05)	
	%MEHP4
	ICC	0.60	NA^a^
	Subject (SE)	0.45 (0.16)	
	Residual (SE)	0.29 (0.06)	
DnBP/DiBP	MnBP		
	ICC	0.62	0.55
	Subject (SE)	0.90 (0.32)	0.63 (0.23)
	Residual (SE)	0.56 (0.12)	0.50 (0.11)
	MiBP
	ICC	0.54	0.48
	Subject (SE)	1.02 (0.40)	0.73 (0.31)
	Residual (SE)	0.85 (0.19)	0.80 (0.18)
	MCPP
	ICC	0.44*	0.41*
	Subject (SE)	0.56 (0.25)	0.48 (0.22)
	Residual (SE)	0.70 (0.15)	0.69 (0.15)
BBzP	MBzP		
	ICC	0.66	0.65
	Subject (SE)	1.76 (0.60)	1.48 (0.52)
	Residual (SE)	0.91 (0.20)	0.79 (0.17)
DEP	MEP		
	ICC	0.30*	0.21
	Subject (SE)	0.56 (0.34)	0.36 (0.29)
	Residual (SE)	1.33 (0.29)	1.31 (0.29)

NA, not applicable.

a %MEHP is not changed by creatinine adjustment.

* *p* ≤ 0.05 for type 3 test of differences between ≥ 2 samples over 4–6 weeks.

**Table 5:** Sensitivity and specificity (ng/mL) of high/low phthalate exposure classification in a pregnant woman based on a single urine sample compared with 3–5 samples per subject.

		Median (95% CI)	
Phthalate diester	Phthalate metabolite	Specificity	Sensitivity
DEHP	MEHP	0.80 (0.67–0.93)	0.64 (0.38–0.89)
	MEOHP	0.63 (0.44–0.86)	0.60 (0.40–0.88)
	MEHHP	0.50 (0.29–0.70)	0.62 (0.47–0.83)
DnBP/DiBP	MnBP	0.88 (0.72–1.00)	0.67 (0.44–0.89)
	MCPP	0.56 (0.29–0.83)	0.74 (0.60–0.89)
	MiBP	0.95 (0.87–1.00)	0.50 (0.25–1.00)
BBzP	MBzP	0.73 (0.57–0.92)	0.73 (0.54–0.89)
DEP	MEP	0.43 (0.18–0.71)	0.72 (0.56–0.88)
